# Analysis of serum changes in response to a high fat high cholesterol diet challenge reveals metabolic biomarkers of atherosclerosis

**DOI:** 10.1371/journal.pone.0214487

**Published:** 2019-04-05

**Authors:** Biswapriya B. Misra, Sobha R. Puppala, Anthony G. Comuzzie, Michael C. Mahaney, John L. VandeBerg, Michael Olivier, Laura A. Cox

**Affiliations:** 1 Center for Precision Medicine, Department of Internal Medicine, Section of Molecular Medicine, Wake Forest School of Medicine, Medical Center Boulevard, Winston-Salem, North Carolina United States of America; 2 Department of Genetics, Texas Biomedical Research Institute, San Antonio, Texas, United States of America; 3 The Obesity Society, Silver Spring, Maryland, United States of America; 4 South Texas Diabetes and Obesity Institute and Department of Human Genetics, The University of Texas Rio Grande Valley School of Medicine, Brownsville, Texas, United States of America; 5 Southwest National Primate Research Center, Texas Biomedical Research Institute, San Antonio, Texas, United States of America; Max Delbruck Centrum fur Molekulare Medizin Berlin Buch, GERMANY

## Abstract

Atherosclerotic plaques are characterized by an accumulation of macrophages, lipids, smooth muscle cells, and fibroblasts, and, in advanced stages, necrotic debris within the arterial walls. Dietary habits such as high fat and high cholesterol (HFHC) consumption are known risk factors for atherosclerosis. However, the key metabolic contributors to diet-induced atherosclerosis are far from established. Herein, we investigate the role of a 2-year HFHC diet challenge in the metabolic changes of development and progression of atherosclerosis. We used a non-human primate (NHP) model (baboons, n = 60) fed a HFHC diet for two years and compared metabolomic profiles in serum from animals on baseline chow with serum collected after the challenge diet using two-dimensional gas chromatography time-of-flight mass-spectrometry (2D GC-ToF-MS) for untargeted metabolomic analysis, to quantify metabolites that contribute to atherosclerotic lesion formation. Further, clinical biomarkers associated with atherosclerosis, lipoprotein measures, fat indices, and arterial plaque formation (lesions) were quantified. Using two chemical derivatization (i.e., silylation) approaches, we quantified 321 metabolites belonging to 66 different metabolic pathways, which revealed significantly different metabolic profiles of HFHC diet and chow diet fed baboon sera. We found heritability of two important metabolites, lactic acid and asparagine, in the context of diet-induced metabolic changes. In addition, abundance of cholesterol, lactic acid, and asparagine were sex-dependent. Finally, 35 metabolites correlated (R^2^, 0.068–0.271, P < 0.05) with total lesion burden assessed in three arteries (aortic arch, common iliac artery, and descending aorta) which could serve as potential biomarkers pending further validation. This study demonstrates the feasibility of detecting sex-specific and heritable metabolites in NHPs with diet-induced atherosclerosis using untargeted metabolomics allowing understanding of atherosclerotic disease progression in humans.

## Introduction

About 17.7 million people died from cardiovascular diseases (CVDs) in 2015, representing 31% of all global deaths. Of these, 7.4 and 6.7 million were due to coronary heart disease (CHD) and stroke, respectively [1]. The National Center for Health Statistics (NCHS) data suggested 787,650 deaths (32% of all mortalities) from CVDs with an estimated 83.6 million affected in USA in 2010 alone [2]. CVDs are associated with a wide range of genetic, dietary, environmental, and lifestyle factors; and other risk factors such as hypertension, diabetes, hyperlipidemia, but the majority of these have not been fully characterized.

Atherosclerosis is a chronic inflammatory disorder in arteries which causes major clinical problems such as acute myocardial infarction and stroke. Atherosclerotic plaques are characterized by an accumulation of macrophages, lipids, smooth muscle cells, and fibroblasts, and, in advanced stages, necrotic debris within the arterial wall. Among cardiometabolic risk factors, elevated serum cholesterol can induce development of atherosclerosis in the absence of other risk factors [3]. To assess the impact of diet on atherosclerosis, the Lifestyle Heart Study randomized patients with coronary atherosclerotic heart disease to a low-fat vegetarian diet or a standard diet for one year. Patients on the low-fat vegetarian diet showed regression of angiographically detected coronary atherosclerosis and a 91% reduction of frequency of angina, while patients on the standard diet showed a 186% increase in the frequency of angina [4, 5]. Similarly, long-term caloric restriction in humans has been shown to have a powerful protective effect against atherosclerosis when compared to subjects on a normal American diet [6]. Many diets and dietary components have been shown to impact coronary artery disease, including the protective effect of the Mediterranean diet, the “prudent” diet, vegetables, and nuts, or the harmful effect of a “Western” diet (WD), trans-fatty acids, and a diet with a high glycemic index or load [7]. The WD is also known to exacerbate the metabolic syndrome, obesity, and diabetes, in addition to atherosclerosis.

The plasma levels of apoB, apoAI, remnant-like particle (RLP)-C, lipoprotein (a) [Lp(a)], and hs-CRP, in addition to plasma levels of lipids, are typically used as predictors of atherosclerosis in clinical practice [8], whereas non- high-density lipoprotein cholesterol (HDL-C) is a better predictor of severity of coronary atherosclerosis than LDL-C [9]. Unfortunately, the visit-to-visit LDL-C and HDL-C in a 5-year follow-up study (n = 130 patients) presented with ST-segment elevation myocardial infarction (STEMI) [10] was shown to be highly variable. Moreover, a large meta-regression analysis indicated that an increased amount of circulating HDL-C alone does not reduce the risk of CHD events, CHD deaths, or total deaths [11]. HDL-C does not necessarily reflect HDL function, and HDL function may be a better biomarker of cardiovascular risk [12]. Thus, the underlying complex metabolic changes in serum correlated with diet-induced atherosclerosis remain unknown. Additional serum-based biomarkers with higher accuracy in reflecting these complex changes potentially would be useful for human diagnostic testing of atherosclerosis.

The diversity of environmental and genetic factors that contribute to atherosclerosis results in extensive phenotypic variation and consequently in individual serum metabolomes. A typical GC-TOF-MS -based metabolomics study can quantify nearly 80 metabolites in serum [13]. Studies have revealed significant changes in the human serum metabolome one minute after a cardiac ischemic event [14]. However, no metabolites have been associated with atherosclerosis that could provide more accurate biomarkers than current serum-based markers such as LDL-C. A pedigreed cohort of baboons provide a unique opportunity to explore the heritability of these circulatory serum metabolites with the ability to control environment and genetics.

Interestingly, atherosclerosis risk, and the risk for CVD complications, is different in men and women. In a study involving 142 coronary artery disease patients, men had more severe structural and functional abnormalities in epicardial coronary arteries than women, even in patients with early atherosclerosis [15]. Another study spanning 12 years, where 495 cases of first myocardial infarction among men and 103 cases among women revealed that myocardial infarction incidence was 4.6 times higher among men [16]. Finally, another study involving 1782 subjects at risk of CVD including 926 cases with hypercholesterolemia, revealed that important sex differences exist in the current clinical definitions of the metabolic syndrome with regard to predicting early atherosclerotic lesions [17]. Moreover, the sex differences in lipid and lipoprotein metabolism have been attributed to a variety of factors, not just sex hormones [18], but sex-specific differences affecting metabolite levels, such as sex dimorphism in cholesterol kinetics, remain to be elucidated. Women exhibit more favorable adipokine, lipid, and immune profiles compared to men, which may explain the lower instability grade in their carotid atherosclerotic plaques [19].

For over 50 years, baboons have served as an experimental non-human primate (NHP) model for cardiometabolic disorders, owing to their commonalities in genome, pathophysiology (in cardiovascular diseases, obesity, type 2 diabetes), and development to humans [20, 21]. In human biomedical research, where controlled diet-challenge studies suffer from logistic drawbacks and other confounding factors (diet dysregulation, life styles, ethnicity-genotype issues etc.), and obtaining tissue biopsies from healthy individuals, is challenging, studies with NHPs circumvent these limitations. In captivity, controlled and long-term diet-challenge studies in baboons are feasible. The baboon is a well-characterized model for dyslipidemia and atherosclerosis [22, 23], and is very similar to humans both physiologically and genetically. More recently, research efforts have focused on understanding atherosclerosis using epigenetics [24], gut microbiome [25], miRNA [26], DNA methylation, and mitochondrial roles [27] among other approaches. Baboons on a HFHC diet develop typical cardiometabolic diseases, such as obesity, insulin resistance, dyslipidemia, atherosclerosis, and CVD, essentially identical to those of humans. In a recent analysis, we have demonstrated characteristic transcriptomic (mRNA and miRNA) changes in HFHC diet-challenged baboons [28, 29].

Previously, Barba and colleagues [30] used nuclear magnetic resonance spectroscopy (^1^H NMR) based metabolomics analysis of blood serum to discriminate patients with myocardial ischemia by changes in lactate, glucose, and saturated lipid resonances following exercise. Similarly, using untargeted LC-MS based metabolomics in venous blood collected from human subjects with coronary atherosclerosis, Gao et al [31] suggested significant metabolic dysfunction in phospholipid, sphingolipid, and fatty acid metabolism in the patients. In another study, using ultra-high performance liquid chromatography coupled to quadruple time-of-flight mass spectrometry (UHPLC-QTOF/MS), Li et al [32] explored the global metabolic perturbation profile for CHD in 300 human subjects and identified 3 novel metabolites (4-pyridoxic acid, PG (20:3/2:0) and lithocholic acid) that exhibited strong correlations with CHD. Moreover, the above studies captured many fatty acids and lipid metabolites, but not polar metabolites which are only captured in GC-MS-based analyses. In addition, none of the above studies was conducted under controlled dietary conditions, which are difficult in research with human subjects.

Here, we report on characterization of circulatory serum metabolite changes in HFHC diet-challenged baboons that are associated with atherosclerotic disease status. The identification of metabolite-based biomarkers will strengthen our understanding of atherosclerosis development and progression. Studies have revealed significant changes in the human serum metabolome one minute after a cardiac ischemic event [14]. However, no metabolites have been associated with atherosclerosis that could provide more accurate biomarkers than current serum-based markers such as LDL-C. Analysis of our pedigreed cohort of baboons provides a unique opportunity to understand the heritability of these circulatory serum metabolites in the context of HFHC diet challenge. In addition, identification of individual metabolites and pathway biomarkers of atherosclerosis will help improve diagnosis of atherosclerosis beyond current clinical practice.

## Materials and methods

### Chemicals

Acetonitrile, isopropanol, methanol, and pyridine (all HPLC grade solvents), and methoxyamine hydrochloride (MeOX), 1% TMCS in *N*-methyl-*N*-trimethylsilyl-trifluoroacetamide (MSTFA), 1% TMCS containing N-(t-butyldimethylsilyl)-N-methyltrifluoroacetamide (MTBSTFA), and adonitol (internal standard) were obtained from Sigma-Aldrich, St. Louis, USA.

### Animals and serum samples

All procedures involving animals were reviewed and approved by the Texas Biomedical Research Institute’s Institutional Animal Care and Use Committee (IACUC). Southwest National Primate Research Center (SNPRC) facilities at the Texas Biomedical Research Institute and the animal use programs are accredited by Association for Assessment and Accreditation of Laboratory Animal Care International (AAALAC), operate according to all National Institutes of Health (NIH) and U.S. Department of Agriculture guidelines, and are directed by doctors of veterinary medicine. All animal care decisions are made by the SNPRC veterinarians. All animals were housed in group cages allowing them to live in their normal social groups with *ad lib* access to food and water. Enrichment was provided on a daily basis by the SNPRC veterinary and behavioral staff in accordance with AAALAC, NIH, and U.S. Department of Agriculture guidelines. For this study, we utilized a cohort of olive baboons (*Papio hamadryas*; Taxonomy ID 9557) maintained as part of the baboon colony at the Southwest National Primate Research Center (SNPRC), located on the campus of the Texas Biomedical Research Institute, San Antonio, Texas. The baboons were raised and maintained on a standard monkey chow diet [(high complex carbohydrates; low fat (“Monkey Diet 15%/5LEO,” LabDiet, PMI Nutrition International, St. Louis, MO)] prior to the study initiation. Freshly collected serum samples were stored in aliquots at -80°C until analysis. After study completion animals were euthanized by an SNPRC veterinarian using ketamine and pentobarbital followed by exsanguination in accordance with American Veterinary Medical Association (AVMA) guidelines [33]. Humane endpoint was confirmed by lack of pulse, breathing, corneal reflex, and response to firm toe pinch, and inability of the veterinarian to hear respiratory sounds and heartbeat by stethoscope.

### Study design

In our previously published study [34] 112 baboons from the SNPRC pedigreed colony were fed a HFHC diet for 2 years. Age- and sex- matched young adult baboons (8–12 years) (*Papio hamadryas*) were fed monkey chow *ad libitum* from weaning until the diet challenge study began (Chow diet: 12% energy from fat, 18% from protein, and 69% from carbohydrate consisting of 0.29% glucose and 0.32% fructose; “Monkey Diet 25/50456,” LabDiet, PMI Nutrition International, St. Louis, MO), and were then challenged for 2 years with an atherogenic diet (40% of calories as fat and 1.7 mg of cholesterol per kcal) prepared from a mix of lard, cholesterol, sodium chloride, vitamins [ascorbic acid and vitamin A (retinyl acetate)], and water to a base diet (“Monkey Diet 25/50456,” LabDiet, PMI Nutrition International, St. Louis, MO). The approximate mean per animal daily intake was 400 g (~1200 kcal) for the HFHC diet. Clinical measures were obtained from plasma samples for the described cohort of baboons at the baseline (chow diet) and at the end of the 2-year HFHC diet challenge. These included body weight, lipoprotein measures, lesion data, inflammation and oxidative stress markers, and arterial compliance. For this particular study, we selected a subset of those 112 animals (males = 34, females = 26) based on the values for blood concentrations of LDL-C, HDL-C, total cholesterol, and glucose, and atherosclerotic lesion burden, ectopic fat scores for heart, and body weight as the end-point measures to select the most extreme HFHC diet “responders” and “non-responders”, i.e., to enrich the extreme metabolic phenotypes and thus to enhance the ability to discern signal (where large scale changes in clinical measures were observed) from noise (where no significant changes in clinical measures were recorded).

### Arterial lesions, CVD-related biomarkers, lipoprotein and other clinical measures

Lesion formation in three major arteries: the aortic arch, thoracic section of the descending aorta, and the common iliac artery were assessed in baboons after 2 years of HFHC diet challenge that were humanely euthanized and were subjected to standard necropsy procedures as described [34]. Total serum cholesterol and triglyceride (TG) concentrations were determined enzymatically using commercial reagents in a clinical chemistry analyzer. HDL-C was measured in the supernatant after heparin- Mn^+2^ precipitation. Blood glucose was measured using the Alfa Wasserman ACE clinical chemistry instrument (West Caldwell., NJ). Summaries of the raw data are presented as means, and standard deviations.

### Serum sample extraction and derivatization for GC-MS analysis

Aliquots (30 μL) of serum samples were subjected to sequential solvent extraction, once each with 1 mL of acetonitrile: isopropanol: water (3:3:2) and 500 μL of acetonitrile: water (1:1) mixtures at 4°C [35]. Adonitol (5 μL from 10 mg/ml stock) was added to each aliquot as an internal standard prior to the extraction. The pooled extracts (~ 1500 μL) from the two steps were dried under vacuum at 4°C prior to chemical derivatization (silylation reactions). Dummy extractions performed in microcentrifuge blank tubes served as extraction blanks to account for background noise and other sources of contamination. Serum samples were then sequentially derivatized with methoxyamine hydrochloride (MeOX) and 1% TMCS in *N*-methyl-*N*-trimethylsilyl-trifluoroacetamide (MSTFA) or 1% TMCS containing N-(t-butyldimethylsilyl)-N-methyltrifluoroacetamide (MTBSTFA) as described elsewhere [36–39]. Briefly, the steps involved addition of 10 μL of MeOX (20 mg mL^-1^) in pyridine incubated under shaking at 55°C for 60 min followed by trimethylsilylation at 60°C for 60 min after adding 90 μL MSTFA or MTBSTFA [38–40].

### Metabolomic analysis by 2D GC-ToF-MS

Two dimensional gas chromatography-mass spectrometry (2D GC-ToF-MS) was performed as described [36]. Derivatized samples were injected in splitless mode using an autosampler (VCTS, Gerstel™, Linthicum, MD, USA) that consisted of an Agilent^©^ 7890 B gas chromatograph (Agilent Technologies, Palo Alto, CA, USA) in line with a Pegasus ® 4D ToF-MS instrument (Leco Corp., San Jose, CA, USA) with an electron ionization (EI) source. Injection temperature was set at 250°C (front inlet) and helium at 1 mL min^-1^ was used as a carrier gas. Separation on the GC was achieved using two columns in line, a primary Rxi®-5Sil MS capillary column (Cat. No. 13623–6850, Restek, Bellefonte, PA, USA) (30 m × 0.25 mm × 0.25 μm) and a secondary Rxi®-17Sil capillary column (Cat. No. 40201–6850, Restek, Bellefonte, PA, USA) (2 m × 0.15 mm × 0.15 μm). The temperature program for the primary column started isothermal at 70°C for 1 min followed by a 6°C min^-1^ ramp to 310°C and a final 11 min hold at 310°C. The secondary oven temperature was programmed with an offset of 5°C; the modulator temperature offset was 15° C relative to the first oven temperature. The modulation temperature (second-dimension separation time) was set at 4 s divided into a hot and cold pulse times of 0.60 s and 1.4 s, respectively between the two stages. Transfer line temperature was maintained at 250°C and the ion source temperature was 250°C. All samples were run with an offset time of 470 s to allow the solvents and derivatizing reagents to pass through without reaching the detector. The system was then temperature-equilibrated at 70°C for 5 min before the next sample. The acquisition sequence started with blank solvent (pyridine) injections, followed by a randomized list of the following: extraction blanks (B), reagent blanks (R), and samples (S). Further, pooled QC samples were injected at scheduled intervals for tentative identification and monitoring shifts in retention times for quality control (QC) checks. Mass spectra were collected at 200 scans/s with a range of *m/z* 40–600.

### Processing of 2D GC-MS data

The 2D GC-MS data sets were processed (cleaned, aligned, and annotated) using ChromaToF version 4.71.0.0 (LECO Corp., Michigan, USA) as described [36, 38, 39] with settings such as S/N: 25; peak width: 0.15, base line offset: 1; m/z range: 40–600, library matching score cut off at 600 as initial cut-off). Spectral library matching for compound identification was performed essentially following settings as described earlier [38, 39]. The filtered raw GC-MS data included ~600 manually curated metabolites. Base peak areas (BPCs) of the mass fragments (*m/z*) were normalized using median normalization followed by log_2_ transformation. Peak areas were further normalized by dividing each peak area value by the area of the internal standard (adonitol) for a given sample. For both platforms, metabolite annotation and assignment followed the metabolomics standards initiative (MSI) guidelines for metabolite identification [41], i.e., Level 2: identification was based on spectral database (match factor >80%) which is accepted for EI-MS mass spectra generated at 70 eV and Level 3: only compound groups were known, e.g. specific ions and RT regions of metabolites leading to putative identification.

### Statistical analysis

Statistical processing of both the GC-MS data sets was performed using statistical software R (Version 3.5.1) [42, 43]. Normalized, transformed, imputed, outlier removed, and scaled peak area representative of relative metabolite amounts obtained from using DeviumWeb [44] are presented. We used fold change (FC) cut offs of FC < 0.8 as low and FC > 1.2 as high as arbitrary values to find directionality of change for diet (chow vs HFHC) and sex (males vs females). All P-values reported were nominal with a cut-off for significance (P, < 0.05) unless specified otherwise.

### Univariate and multivariate analysis

Hierarchical clustering analysis (HCA) was performed on Pearson distances using the standalone tool PermutMatrix [45], where the data was normalized using z-scores of the relative abundance of the metabolites for heat map display. Correlations were performed using R package ‘*corrplot*’ and reported are Pearson and Spearman rank correlations. Principal components analysis (PCA) was performed using DeviumWeb [44] where the output displayed score plots to visualize the sample groups. The data were scaled with unit variance without transformation.

### Pathway enrichment and clustering analysis

Pathway enrichment analysis was performed using the webserver MetaboAnalyst 3.0 (www.metaboanalyst.ca) [46]. Pathways were connected as networks and visualized using Cytoscape (version 3.6.1) for visualization purposes. For ID conversions, the Chemical Translation Service (CTS: http://cts.fiehnlab.ucdavis.edu/conversion/batch) was used to convert the common chemical names into their Kyoto Encyclopedia of Genes and Genomes (KEGG), Human Metabolome Database (HMDB), METLIN, PubChem Compound ID (PubChem CID), and Chemical Entities of Biological Interest (ChEBI) identifiers.

### 2D GC-ToF- MS–based raw metabolomics datasets availability

The raw datasets and the metadata obtained from mass-spectrometry based 2D GC-ToF- MS metabolomics efforts are made publicly available at the deposited at the Global Natural Product Social Molecular Networking (GNPS) database (Study ID: MassIVE MSV000083256) and is available for download at this link: ftp://massive.ucsd.edu/MSV000083256 (doi:10.25345/C5Z608) as .cdf (netCDF) files.

### Heritability estimates

We used a maximum-likelihood-based variance decomposition approach implemented in Sequential Oligogenic Linkage Analysis Routines (SOLAR) [47] to estimate heritability for each metabolite in the 60 pedigreed baboons maintained on a chow diet and HFHC diet. This approach uses information from possible relationships (i.e., kinship) simultaneously to disentangle the genetic architecture of the metabolites. In a simple model, variances or covariances between relatives as a function of the genetic relationships can be specified, and the proportion of phenotypic variance that is attributed to (additive) genetic effects [i.e., heritability (*h*^2^)] can be estimated from the components of variance [47]. The *P* values for the heritability estimates are obtained by likelihood ratio tests, where the likelihood of a model is estimated and compared with the likelihood of a model in which the heritability is constrained to zero. Two times the difference in the natural logarithmic likelihood is distributed asymptotically as a 1/2:1/2 mixture of a *χ*^2^ variable with one degree of freedom and a point mass at zero [48].

## Results and discussion

A recent study in a cohort of 112 baboons showed that lipoprotein measures are associated with atherosclerotic lesion burden in this NHP [34]. We used an untargeted MS-based metabolomics approach in in a subset of this cohort to identify circulating serum metabolites in baboons before (baseline, 0 d) and after a 2-year HFHC diet challenge in this model of atherosclerosis. This long-term diet-challenge has the potential to allow identification of heritable metabolites predictive of atherosclerosis, yield serum-based metabolite biomarkers of atherosclerotic lesion burden, and provide novel biomarkers for early detection/diagnosis of atherosclerosis.

### Clinical measures and fat scores as a result of diet-induced changes

**Fig 1:**
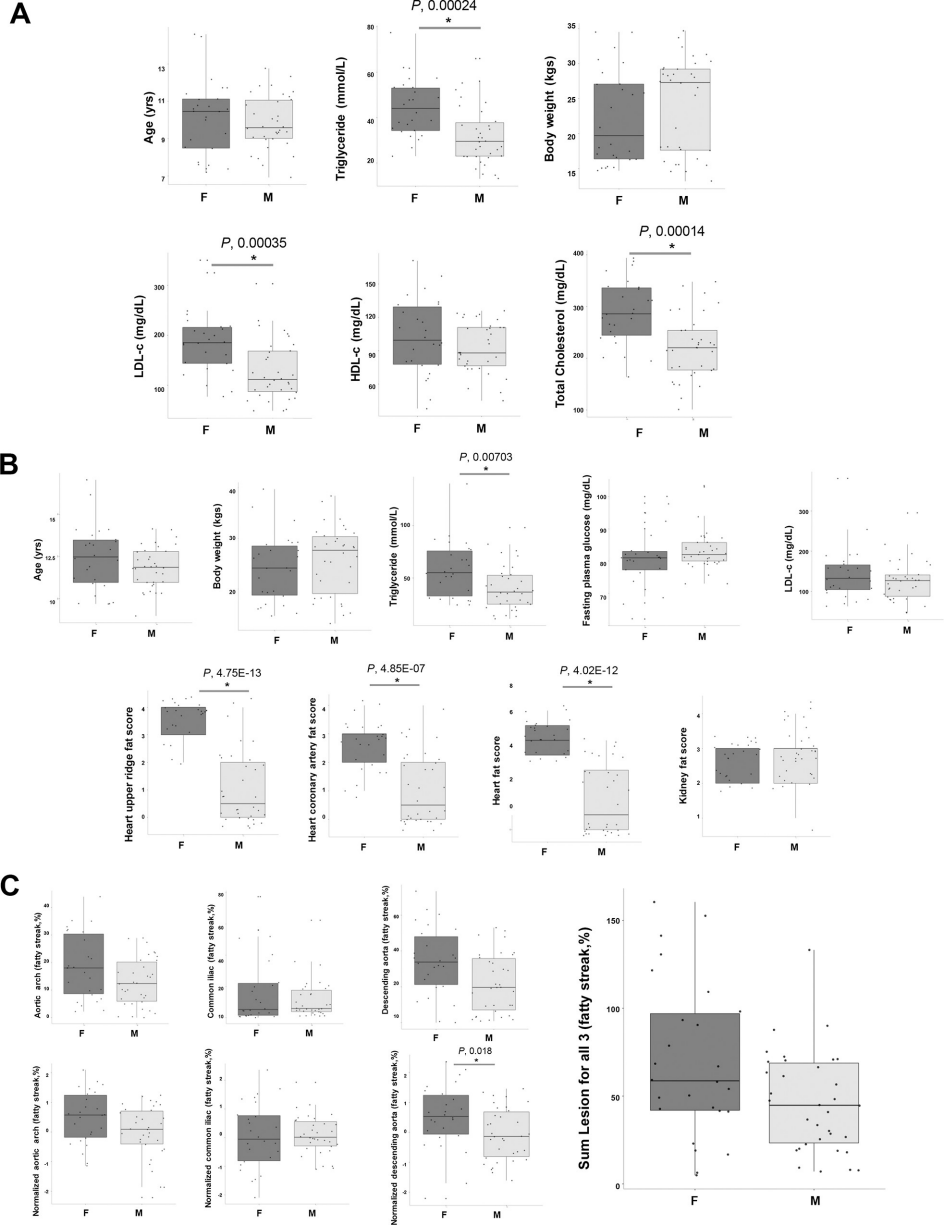
Changes in clinical measures of 60 animals, and fat score indices for the males (n = 26) and females (n = 34) at [A] baseline and at [B] end of the 2 yr diet challenge. Also provided are the **[C]** lesion data for the 2 yr diet-intervention. Values are provided as mean +/- standard error of mean (SEM). Analysis performed was a nonparametric two-tailed t-test, comparing proportion normalizing with proportion between males and females and chow and HFHC diet intervention. *P < 0*.*05*. In the box-plots, the center lines show the medians; box limits indicate the 25^th^ and 75^th^ percentiles as determined by statistical software R; whiskers extend 1.5 times the interquartile range from the 25^th^ and 75^th^ percentiles, outliers are represented by dots; crosses represent sample means; data points are plotted as black dots. Widths of boxes are proportional to square-roots of the number of observations.

In the 60 baboons at the baseline (0 d), we observed no significant differences (P, >0.05) in age, body weight (BW), and HDL-C among male (n = 26) or female (n = 34) baboons. Further, we observed that BW (P, 6.044E-06), glucose (P, 0.0303), LDL-C (P, 3.113E-21), and TG (P, 0.00003) levels were all significantly different for baseline (0 d) by comparison with HFHC diet (2 yr) baboons (**Table 1; S1 Table**). In addition, significant differences between males and females were observed for TG (P, 0.00024), LDL-C (P, 0.00035), and total cholesterol (P, 0.00014) (**Fig 1A**).

**Table 1:** Comparisons of changes from baseline (0yr, Chow diet) to treatment (2 yrs, HFHC diet) in clinical measures between the two treatments (diet) groups.

Factor	Chow Diet	HFHC Diet	T-test (P-value)
**No of baboons**	N = 60	N = 60	-
**BW (KGs)-** **Mean, 95% CI**	23.19 (21.50,24.88)	25.02 (17.82,32.23)	6.044E-06
**Glucose (mg/dL)-** **Mean, 95% CI**	78 (75.25,80.74)	82.18 (75.44,88.92)	3.033E-02
**LDL-C (mg/dL)-** **Mean, 95% CI**	43.41 (27.41,59.41)	133.41 (117.26,149.56)	3.113E-21
**TG (mg/dL)-** **Mean, 95% CI**	37.33 (31.97,42.69)	48.23 (41.86,54.60)	3.067E-04

All P-values were significant, P, 0.05.

Indeed, in the original study with 112 baboons, the concentrations (or activities) of the majority of circulating biomarkers of lipid/lipoprotein metabolism and inflammation showed mean changes in the animals fed the HFHC challenge diet for 2 years, but the values for HDL-C and LDL-C at 2 years (week 104) were not significantly different from those at the baseline (week 0) [34]. At the end of the 2-year diet challenge, fat scores for heart upper ridge, heart, coronary artery, and kidney were assessed (**Fig 1B; S1 Table**). We noted, significant changes in TG (P, 0.00703) and fat scores such as heart upper ridge fat score (P, 4.75E-13), heart coronary artery fat score (P, 4.85e-07), and heart total fat score (P, 4.02E-12), all of these which fat scores were significantly higher for females than for males.

Fatty streaks are typically characterized by enhanced accumulation of non-foamy and foamy macrophages with a small amount of extracellular lipids [49], that form even at relatively low-level hypercholesterolemia (200 to 400 mg/dl) in NHPs [50]. Thus, we analyzed the arterial lesion data obtained from the three major arteries, as fatty streak percentage (%). Our results revealed that the fatty streak percentage was significantly higher only in the descending aorta (P, 0.018) of females (**Fig 1C**). Indeed, epidemiological studies of human populations support experimental studies showing association between high blood cholesterol levels and fatty streaks [51]. Our observations of significantly higher fat scores, TG, total cholesterol, and LDL-C in female baboons were interesting—cholesterol levels at baseline and fatty streaks at the end of the challenge were significantly higher in females than males. In humans, a meta-analysis of 37 prospective cohort studies, the risk of fatal CHD is 50% higher in women with diabetes compared to males with diabetes [52]. Moderate or borderline hypertension (<140/90 mmHg) causes more endothelial dysfunction and cardiovascular complications in women than men [53]. Although women have often been under-represented in statin trials in the past, there is currently no doubt that in secondary prevention LDL-C reduction in women leads to equally lower coronary heart disease mortality as in men [54]. Microvascular dysfunction and diffuse coronary atherosclerosis without obstructive lesions is more prevalent in women than men and can be better visualized with positron emission tomography (PET) and cardiovascular magnetic resonance (CMR) techniques [55]. Beyond NHPs and human studies, the testicular feminized mouse (which has non-functional androgen receptor and low testosterone) develops fatty liver and aortic lipid streaks on a high-fat diet; whereas, androgen-replete XY littermate controls do not [56]. With fatty streak % results showing the varying degree of lesion formation in these HFHC diet fed baboons (**S2 Table**), we proceeded to quantify circulatory metabolites in the serum to correlate with the extent of atherosclerosis.

### Metabolic pathways captured and individual metabolites changing with diet

In our study of the NHP model of atherosclerosis, we used the 2D GC-ToF-MS platform and dual derivatization strategies (MSTFA and MTBSTFA) to quantify 515 metabolites (of which 180 were assigned HMDB IDs) out of the total 3154 metabolite peaks that were found in at least in 2/3^rd^ of the biological samples from the combined two time points (**S1 Fig**). The raw mass spectrometry abundance data (**S3 Table**) were normalized (**S4 Table)** and used for all statistical analysis. After removal of redundant metabolites and conversion to their biological identifiers (KEGG and HMDB), the 321 metabolites were mapped to 71 different KEGG-based pathways such as ammonia recycling, urea cycle, alanine metabolism, phenylalanine and tyrosine metabolism, aspartate metabolism, carnitine synthesis, malate-aspartate shuttle (all significant, P, <0.1), followed by glutamate metabolism, beta-alanine metabolism, propanoate metabolism, glucose-alanine cycle, fatty acid biosynthesis, vitamin K metabolism, arginine and proline metabolism, glycine and serine metabolism, valine, leucine and isoleucine degradation, cysteine metabolism, and oxidation of branched chain fatty acids (not-significant) (**S2 Fig)**. Furthermore, a handful of metabolites tentatively identified could be of exposomal origin (i.e., diet, environment, drugs, hydrocarbons etc.) or are contaminants (**S4 Table**), for which we have yet to associate with disease status. More specifically, all quantified metabolites reported in this study remain to be validated (annotation level 4) to improve confidence in the data using other orthogonal technologies such as LC-MS and NMR [57].

Firstly, using two-way ANOVA analysis for sex and diet, we observed 35 and 34 metabolites that were significantly different for sex and diet, respectively (**S5 Table).** Important metabolites that varied with HFHC diet include N-formyl glycine (P, 0.000449, 0.5 fold), malonic acid (P, 0.009446944, 1.7 fold), aspartic acid (P, 0.0146, 0.6 fold), cholesterol (P, 0.0269, 1.7 fold), propionic acid (P, 0.0321, 1.3 fold), glyoxylic acid (P, 0.0332, 0.5 fold), 3-phenyllactic acid (P, 0.0371, 0.3 fold) among others. Significantly different metabolites for sex (males/females) were benzoic acid (P, 0.001945, 0.4 fold), methoxycitronellal (P, 0.0022063, 0.7 fold), decanedioic acid/ sebacic acid (P,0.00355, 1.9 fold), citramalic acid (P, 0.00465, 2.4 fold), 2-oxo-butanoic acid/alpha-ketobutyric acid (P, 0.00594, 4.6 fold), tyrosine (P, 0.00602, 2 fold), isobutyrate (P, 0.00686, 0.7 fold), hexanoic acid / caproic acid (P, 0.03953, 1.9 fold) among others (**S5 Table).** Further results from a repeated-measures ANOVA conducted for body weight, sex, age, clinical measures (glucose, HLD-C, LDL-C), and kinship suggested significant changes in seven metabolites. These were 3-cresotinic acid (a salicylic acid derivative), methyl malonate (involved in protein and fat metabolism), malonic acid (involved in malonyl-CoA decarboxylate metabolism), glycine (a non-essential amino acid), arsenous acid (arsenic derivative of possible dietary or environmental origin), 2-ketobutyric acid [involved in glycine, methionine, valine, leucine, serine, threonine, isoleucine, propanoate and C-5 branched dibasic acid (BCAA) metabolism], and 3-methyl-2-oxovaleric acid (an abnormal metabolite that arises from the incomplete breakdown of BCAA). Arsenic derivatives are known to promote cardiovascular toxicity of arsenic and eventually atherosclerosis [58]. Moreover, a deficiency of glycine to sarcosine converting enzyme glycine N-methyltransferase is known to aggravate atherosclerosis in apolipoprotein E-null mice [59]. Significant changes in methylmalonate and malonate are indicative of changes in aminomalonic acid pools that are found in atherosclerotic plaques [60]. Methylmalonate levels serve as an important biomarker in malonic and methylmalonic aciduria that are traced to genetic mutations [61]. Another study investigating changes in urinary metabolites from patients with ischemic heart failure found decreased excretion of TCA cycle intermediates [62], and the succinate precursor methyl malonate [63], indicating the involvement of malonate metabolism in cardiovascular ailments. These results indicate that the HFHC diet induced extensive changes in malonate metabolism (malonate and methylmalonate) and BCAA metabolism (2-ketobutyric acid, and 3-methyl-2-oxovaleric acid). In addition, the HFHC diet induced changes in other metabolites which have not previously been associated with atherosclerosis, such as glycine and 3-cresotinic acid. Validation in an independent cohort, i.e., in humans is required to validate the usefulness of these metabolites as biomarkers of atherosclerosis.

### Effect of diet, sex, and genotype on metabolites (trait/ phenotype) and biomarkers of atherosclerosis

The heritabilities (defined as the proportion of phenotypic variation of a trait that can be attributed to genetic variation) of small metabolites and amino acids have been reported to vary between 23% and 55%, whereas those of lipids and lipoproteins are, respectively, from 48% to 62% and 50% to 76% [64]. Previous studies demonstrated the heritability of metabolite concentrations from stored human red blood cells [65]. Use of this pedigreed baboon cohort provided the opportunity to probe the heritability of the quantified metabolites in serum samples for two diets. Our analysis revealed significant heritabilities on chow diet for lactic acid (83%, h^2^ = 0.83, P, 0.014; n = 58), L-asparagine (52%, h^2^ = 0.52, P, 0.008), and alpha-phenylpropanoic acid (31%, h^2^ = 0.31, P, 0.026) for chow diet (**S6 Table**). For the HFHC diet, we found significant heritabilities for lactic acid (67%, h^2^ = 0.66, P, 0.0056) and L-asparagine (48%, h^2^ = 0.48, P, 0.006) (**S6 Table**). Our findings are consistent with the human studies showing that serum metabolites are heritable and that changes from diet and environment can be quantified. On the basal diet, high heritability of lactate, i.e., 0.83 (residual kurtosis, 0.51) indicates that genetic factors (genotype) contribute 83% of the variation among animals when they are fed the basal diet; whereas, after HFHC-diet exposure, the lowered heritability to 0.67 (residual kurtosis, 0.36) is indicative that on HFHC diet, genes are responsible for 67% of the variation among individuals and environmental factors are responsible for 33% of the variation. An East Flanders Prospective Twin Study (of a Belgian population) involving 240 monozygotic and 138 dizygotic twin pairs aged 18 to 34 years revealed that heritability estimates of fasting glucose, fasting insulin, homeostasis model assessment of insulin resistance and beta cell function, as well as insulin-like growth factor binding protein levels, were 67, 49, 48, 62 and 47%, respectively [66]. For total cholesterol, LDL-C, HDL-C and triacylglycerol heritability estimates range between 0 and 98% [66].

In human carotid atherosclerotic plaques, metabolomic profiling revealed enrichment of lactate and taurine, indicative of increased anaerobic glycolytic activity and of inflammatory defense against free radicals, respectively, within the plaques [67]. Indeed, blood lactate is associated with carotid atherosclerosis, as attenuation of the association with adjustment for triglyceride/HDL ratio, a marker of insulin resistance, suggests that lactate's association with carotid atherosclerosis may be related to insulin resistance [68]. Lee et al., [69] performed metabolomic and lipidomic analysis of male C57BL/6J mice fed 1.25% (w/w) cholesterol and 0.5% cholate (w/w) using NMR and LC-MS and found that sulfur amino acid (SAA) and lipid metabolism were perturbed. This study captured amino acids (alanine, threonine, glutamate, tyrosine, phenylalanine, carnitine, glutamine, leucine, isoleucine, lysine, valine, methionine, N, N-dimethylglycine, cysteine, glycine, homocysteine, cystathionine, serine), sugars (glucose, lactose, mannose), organic acids (lactate, citrate, pyruvate, glycolate, fumarate, formate, acetate), glycerol, carnitine, ceramides, and free fatty acids among others using both NMR and LC-MS platforms as we also observed majority of these in our GC-MS results. Interestingly these mice fed an atherogenic diet (AD) were shown to demonstrate elevated levels of lactate in both serum and heart tissues as revealed from NMR and LC-MS analysis [69]. The enzymatic asparaginase treatment (to deplete systemic asparagine) is known to induce clots, strokes, and other thromboembolic events [70], indicating interference with lipid metabolism.

**Fig 2:**
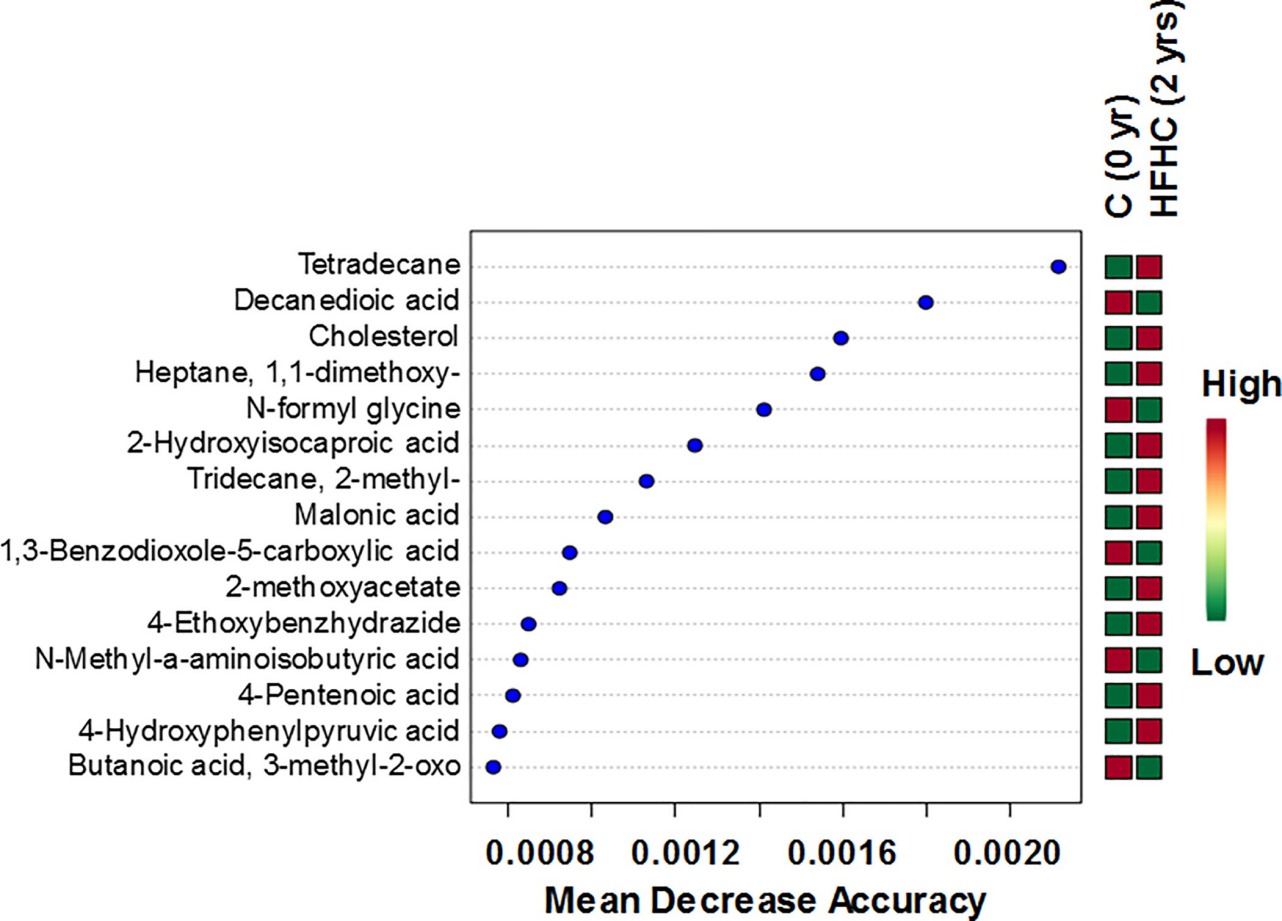
Random forest analysis showing discriminating biomarkers of diet-challenge in their decreasing orders of mean decrease accuracies (MDA). Smaller MDAs tend to be better discriminatory markers than the higher values.

We further probed which metabolite abundance levels allowed discrimination of baseline (0 d) and HFHC diet-challenged (2 yr) serum samples. Random forest analysis is a data-driven method designed for prediction, and is conducted to identify metabolites that improved prediction of ASD and C groups. Contributions of individual predictors are measured by ‘variable importance’ using a conditional permutation scheme for correlated predictors. Variable importance greater (less) than 0 suggests an increase (decrease) in prediction accuracy. By relying on the ranges of values of each selected feature using our RF classifier, we can identify dependencies between features which result in separation for the two classes of interest to help identify the most important metabolites and exclude associations by chance. RF algorithms [71] have been extremely successful as a general purpose classification and regression method, shown to be useful for small sample sizes, and successfully implemented in the metabolomics literature [72–74]. We obtained larger mean decrease accuracy (MDA) values for metabolites involved and associated with fatty acid metabolism, such as cholesterol, decanedioic acid (involved in carnitine-acylcarnitine translocase deficiency and medium chain acyl-CoA dehydrogenase deficiency), 1,1,-dimethoxyheptane (an acetal, which is an energy source or storage molecule), 4-pentenoic acid (a straight chain fatty acid), and large-scale changes in amino acid metabolism, i.e., N-formylglycine (an alanine derivative), 2-hydroxyisocaproic acid (a BCAA amino acid leucine metabolite), N-methyl-alpha-aminoisobutyric acid, 4-hydroxyphenylpyruvic acid (involved in aromatic amino acid biosynthesis), 3-methyl-2-oxobutanoic acid (involved in valine, leucine, and isoleucine biosynthesis/degradation), and organic acids such as 2-methoxyacetate (a carboxylic acid nutrient), malonic acid (a dicarboxylic acid), 1,3-benzodioxole-5-carboxylic acid (an antioxidant), N-Methyl-a-aminoisobutyric acid (involved with IL-1beta and IL-6 transport) and other alkanes (2-methyl-tridecane, tetradecane) (**Fig 2**).

In atherosclerotic rats, higher decanedioic acid (sebacic acid) acid, a dicarboxylic acid known to produce acetyl-CoA and succinyl-CoA, which belong to important intermediates of the TCA cycle, due to their β-oxidation levels, was reported [75]. Dietary PUFAs reduce atherosclerosis by activating macrophage autophagy and attenuating NLRP3 activation (in mice) [76], and where macrophage metabolism undergoes changes in glycolysis pentose phosphate pathway, inflammation responses, and a defective oxidative phosphorylation among others [77]. These clearly indicate that the HFHC diet perturbed immunometabolism. Although single-gene defects in lipid metabolic pathways account only for a very small portion of familial and sporadic atherosclerosis, multiple genes and environmental factors are known to have a major role in determining elevated lipid levels and risk of atherosclerotic events [78]. Furthermore, in patients with stable atherosclerosis, plasma amino acids were significantly decreased, i.e., lower levels of alanine, aspartate, tyrosine, and serine [79]. Another study found significant associations between plasma branched chain amino acid metabolites and CAD and myocardial infarction [80]. A study comparing cardiac extraction of plasma substrates demonstrated that patients with CAD had decreased extraction of alanine and glutamate/glutamine, even when normalized to any differences in arterial amino acid concentrations [81]. These findings point to pervasive changes in amino acid metabolism, fatty acid metabolism, as well as immunometabolism in plasma of individuals with CAD compared to healthy individuals. Moreover, these individual discriminant metabolites can be used to monitor blood-based changes in metabolism in atherosclerotic individuals.

**Fig 3:**
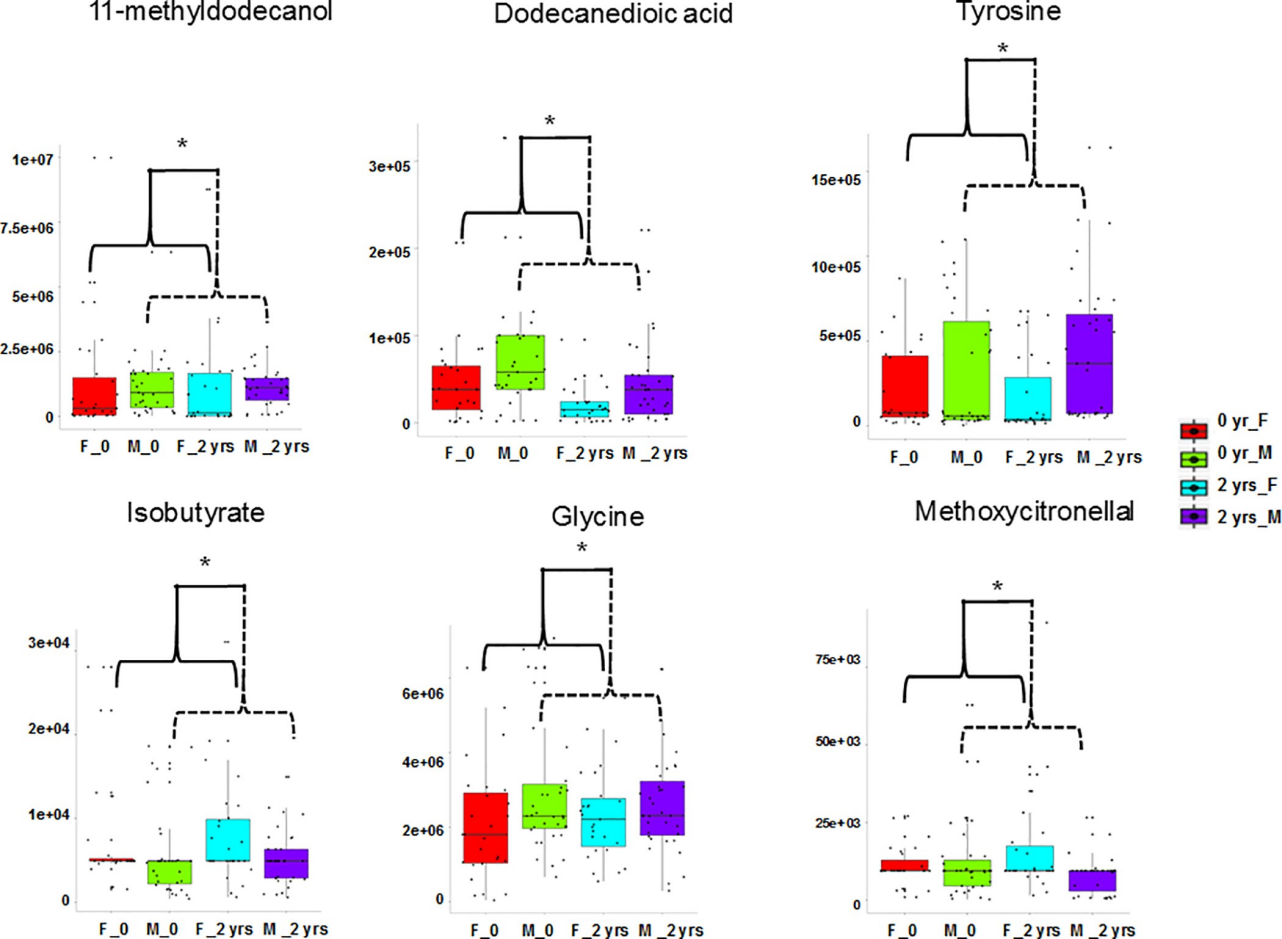
Relative abundances of metabolites that are significantly (ANOVA, P < 0.05) sex-dimorphic. In the box-plots, the center lines show the medians; box limits indicate the 25th and 75th percentiles; whiskers extend 1.5 times the interquartile range from the 25th and 75th percentiles, outliers are represented by dots; crosses represent sample means; data points are plotted as black dots. Widths of boxes are proportional to square-roots of the number of observations.

There is little information in the literature on sex differences in metabolite abundance related to CVD. Lipid metabolism is clearly sex-dimorphic [82]. CAD incidence in a cohort of 14,786 Finnish men and women was ~3 times higher in men compared with women, and mortality was ~5 times higher in men than women [83]. Female macaques, like women, are resistant to atherosclerosis [84]. Our study also revealed sex-dimorphism in abundance of metabolites in baboons (**Fig 3, S5 Table**). For instance, 11-methyldodecanol, decanedioic acid, tyrosine and glycine were significantly (P, <0.05) higher in males; whereas, methoxycitronellal and isobutyrate were significantly (P, <0.05) higher in females, independent of diet. Thus, we observed higher metabolite abundances of amino acids and lipids in females, than in males. Very little information is available on the sex-dimorphic metabolite abundances from human studies, which is starting to be explored and a very new area of active research in metabolomics [85].

### Metabolites associated with clinical measures of atherosclerosis

Increased dietary cholesterol intake is associated with atherosclerosis, and its development requires lipid and inflammatory components [86]. We observed interesting fatty streak lesion variation, including early stage (EGES), flat (F) and raised (R) lesions, corresponding, respectively, to AHA lesion types I, II and V as reported earlier in the original cohort of these baboons [34]. We observed evidence of atherosclerosis in all but one baboon fed the 2‐year challenge diet. CVD risk biomarkers, the prevalence, size, and complexity of arterial lesions, plus consequent arterial stiffness, were increased in comparison with dietary control animals [34]. The subset of 60 baboons used in this diet study were found to have at least one type of lesion.

**Fig 4:**
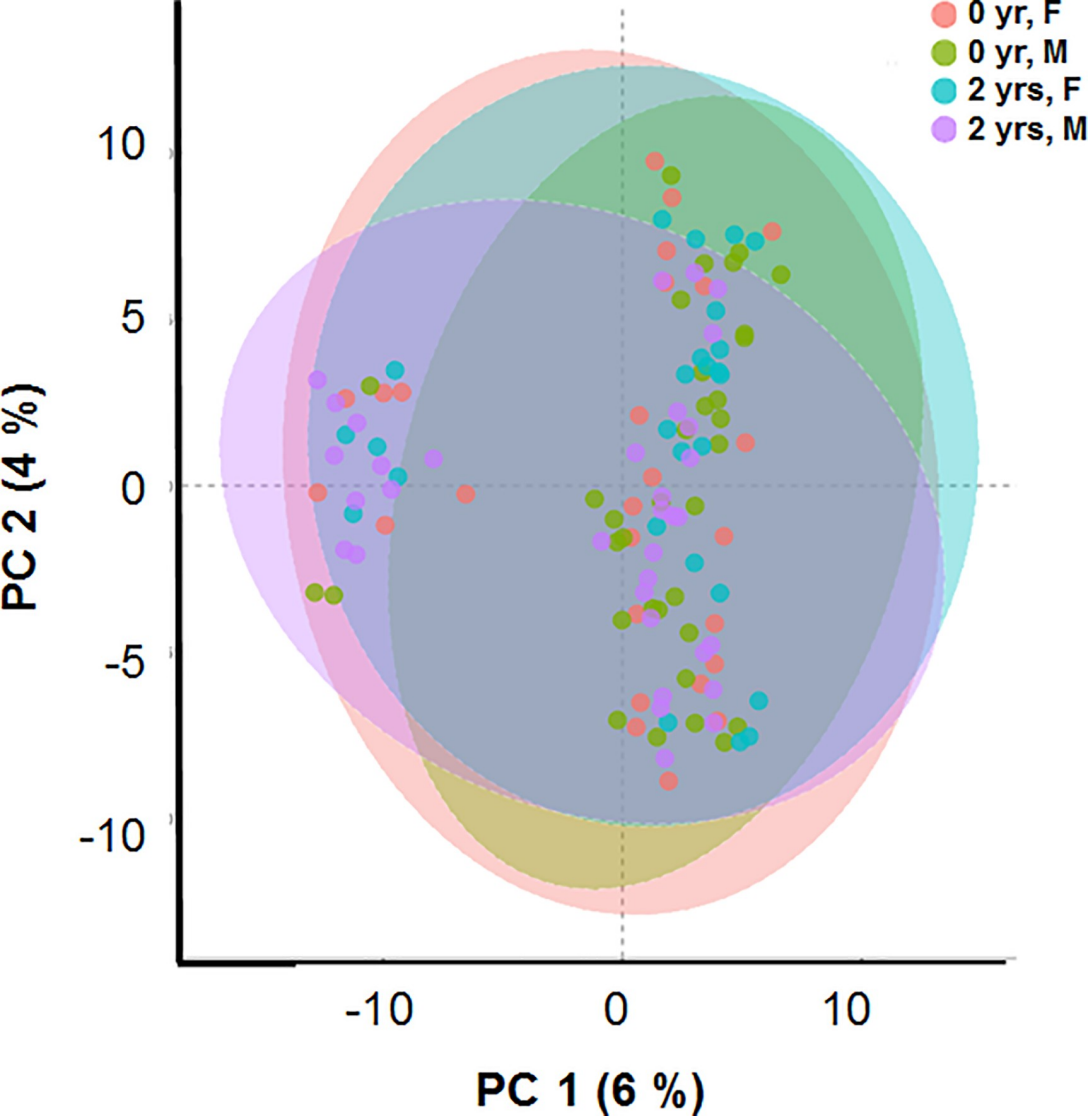
Principal component analysis (PCA) displaying Chow (red) and HFHC diet (cyan) fed baboon samples clustered as well as appearance of a new smaller cluster comprised of 32 baboon samples.

We further probed, whether metabolite abundances at base line (0 d) and post-diet challenge (2 yrs) demonstrated discrimination between these two time points. In the unsupervised PCA score plots, we observed that the two PCs contributed only 10% separation for the two diet-groups (chow and HFHC) (**Fig 4**).

No further improvement of clustering was observed in the partial least square- discriminant analysis (PLS-DA) (i.e., first two PCs contributed only 6%). These analyses point to the fact that the 2 yr diet-induced changes in metabolism are not strong or the metabolites captured were not truly representative of the plaques caused by atherosclerotic events. However, interestingly, a new smaller cluster of 32 serum samples (uniquely represented by 32 baboons) but belonging to both sexes and both diet groups, appeared in this analysis (**S3 Fig**). Upon further interrogation, we observed that these 32 baboons had higher plasma triglyceride (TG) concentrations than the rest of the baboons used in the study (**S3 Table**), which indicates that there must be metabolites, possibly not captured in a GC-MS analysis, in plasma, that are driven by or related to TG metabolism, biosynthesis, and/or accumulation and atherosclerosis. However, we did not detect any significant and notable correlations between plasma TG levels and other metabolites for these baboons or the group as a whole.

**Fig 5:**
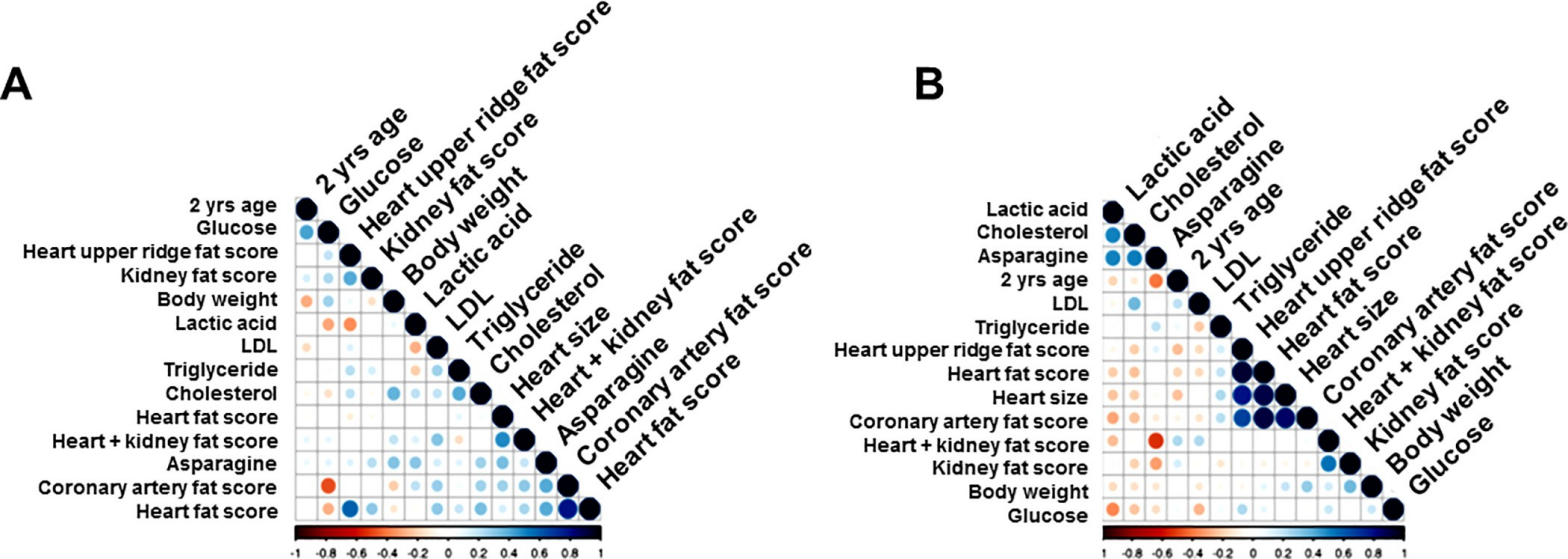
Correlation between fat scores, clinical measures, and selected metabolites in (a) female (n = 26) and (B) male (n = 34) baboons. Correlations are in a scale of +1 (positive, blue) to -1 (negative, red).

Further, we investigated whether clinical measures, heritable metabolites, lesion burden, and fat indices (scores) of various organs were correlated among each other and whether sex played an important role in this correlation. We performed a Pearson correlation analysis with a matrix for fat scores, clinical measures (body weight, LDL-C, TG) with selected metabolites (glucose, cholesterol, lactic acid, and asparagine) separately for males and females (**S7 Table)**. Results demonstrated strong positive correlations among the fat scores from upper ridge, coronary artery, kidney, heart in the males that was weaker in females (**Fig 5**). Indeed, a study in >2100 older men and women in the Rotterdam cohort indicated that presence of carotid atherosclerosis (i.e., carotid intimal–medial thickness of >2.0 mm on carotid screening) was more common among men than women [87]. Location of ectopic fat in key target-organs of cardiovascular control (heart, blood vessels and kidneys) are known to play a role in the pathogenesis of CVD associated with obesity (induced by high-fat diet). Cardiac fat depots within and around the heart impair both systolic and diastolic functions and may in the long-term promote heart failure [88]. Moreover, metabolites lactic acid, cholesterol, and asparagine showed stronger positive correlations among themselves in males than females. However, asparagine correlated with fat scores in females. This shows that plaque-phenotype associated metabolites are well-represented in serum samples, and hence cold be better indicator of disease burden in males than in females. However, interestingly, in other studies metabolite abundance in carotid atherosclerotic plaques were not correlated with metabolite abundance in blood [89].

**Fig 6:**
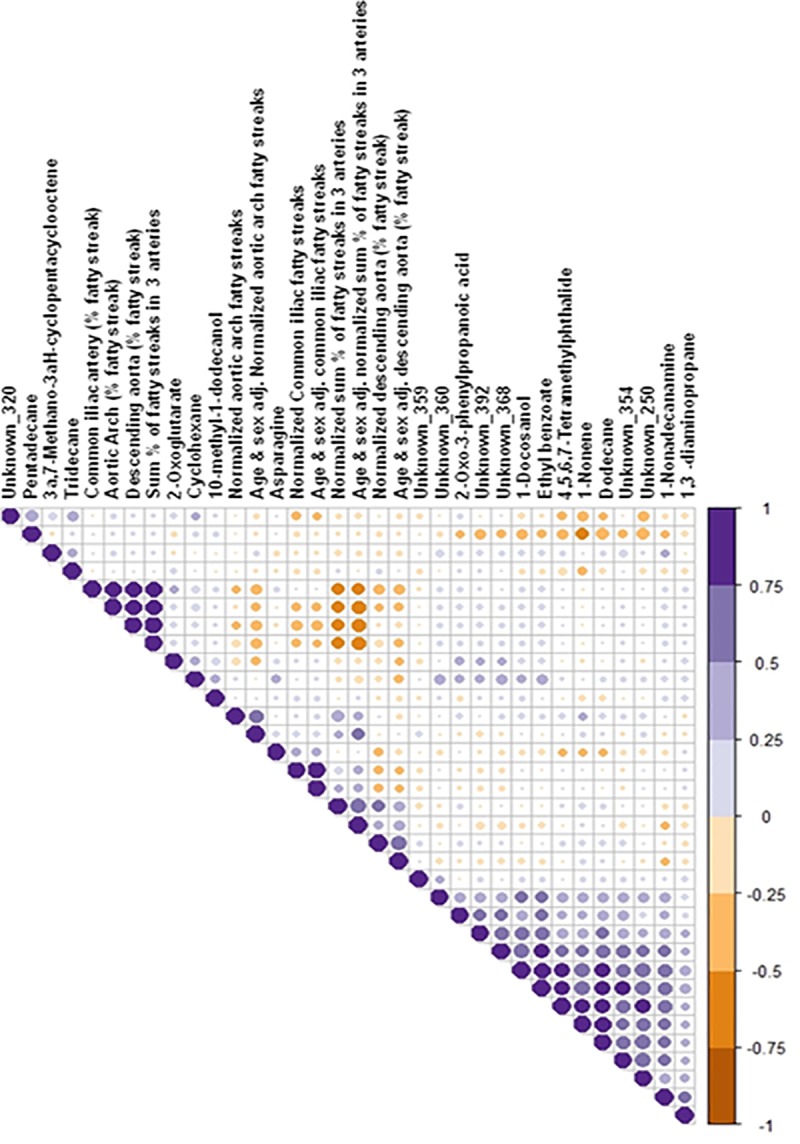
Correlation of atherosclerotic phenotypes (lesion percentage distribution as fatty streak and/ plaque distribution and numbers) with the metabolite abundances. Correlations are in a scale of +1 (positive, purple) to -1 (negative, brown).

We also assessed correlations of metabolite abundance with individual atherosclerotic phenotypes including lesion burden (percent covered by fatty streak or plaques, and the number of plaques) for aortic arch, common iliac artery, descending aorta, and total lesion burden, after adjustment for sex and age (**S8 Table)**. We found that tridecane and 2-oxoglutarate showed strong correlation (R^2^, 0.6–0.8) with all four phenotypes, i.e., aortic arch, common iliac artery, descending aorta, and total lesion burden. Mahaney et al [34], also suggested significant correlations for the aortic arch and common iliac artery for lesion sizes. In addition, we found that asparagine, 2-oxo-3-phenylpropanoic acid, 2-oxoglutarate, and 1, 3-diaminopropane (a polyamine) strongly correlated among each other, but not with lesion burden (**Fig 6)**. We found 35 metabolites significantly correlated (P, <0.05) with total lesion burden including metabolites in pathways involved in ammonia recycling (asparagine, and pyruvate), aspartate metabolism (asparagine, and malonic acid), fatty acid metabolism (palmitic acid, malonic acid), glycolic acid, isobutyric acid, tyramine, methymaleic acid, ethanol. Benzoylformate showed the highest correlation (R^2^, 0.27; P, 0.000035). However, it remains to be seen if the other metabolites identified tentatively as ‘unknowns’ that are correlated with the various lesion and fat score phenotypes are truly metabolites of exogenous origin, or are poorly annotated compounds requiring further structural validation (**S9 Table)**.

### Comparability of the findings in NHP with human studies

Very few studies have been conducted in human atherosclerosis and CVD using metabolomics as a tool for biomarker discovery. Population based studies are laced with confounders such as life style, race, and dietary variations; nevertheless, we probed the overlapping biomarkers using qualitative analysis from recent studies. Vorkas et al [90] investigated the lipid metabolite profile in different areas of the same atherosclerotic tissues. They demonstrated clear lipid heterogeneity within these lesions and identified phosphatidylethanolamine-ceramide as a novel candidate biomarker for atherosclerosis development. Nine metabolites involved in lipid metabolism (i.e. lysophosphatidylcholine (LysoPC) (20:4), LysoPC(16:0), phosphatidylglycerol (18:0/0:0), elaidic acid, prasterone sulfate, l-fucose, monoglyceride (MG) (0:0/18:2(9Z,12Z)/0:0), diglyceride (DG) (20:2(11Z,14Z)/18:3(9Z,12Z,15Z)/0:0), and indoxylsulfuric acid) were selected as the best predictors for early coronary atherosclerosis discrimination [31] using LC-QToF-MS analysis, which are not captured in a GC-MS analysis. Other recent LC-MS and NMR–based studies based on atherosclerotic plaques or plasma samples in humans have shown [91–94] either lipids or other LC-MS amenable metabolites to be important atherosclerosis or CVD biomarkers, i.e., tryptophan, BCAA, gluconate, 2-hydroxycaproate, and trimethylamine n-oxide (TMAO)(a gut-microbiota derived established metabolite). Thus, our study is one the first in diet-induced atherosclerosis that has identified GC-MS amenable metabolites associated with CVD phenotypes.

### Strengths and limitations of the study

For the first time using a dual derivatization strategy and a 2D GC-MS platform for untargeted metabolomic analysis of serum, we showed diet-associated changes in atherosclerotic baboons at baseline and after a 2 yr HFHC diet challenge. Using a pedigreed cohort of NHP with controlled environment, analyses of metabolite abundance, and lesion and fat scores, we identified metabolites correlated with CVD signatures in serum. This model system also allowed us to investigate heritabilities of metabolites in the presence and absence of a HFHC diet challenge. Lastly, the obtained panel of metabolites have the potential to be confirmed in future human studies for replication and validation as biomarkers.

GC-MS as a technology is limited to analysis of polar metabolites and a handful of lipid metabolites. Although using a high throughput 2D platform and two derivatization reagents we analyzed more metabolites than are typically captured in conventional 1D GC-MS, an untargeted LC-MS based approach would have captured diverse metabolic intermediates and non-polar compounds not found in this study. Another interesting scope would be to analyze the metabolite composition of the diet itself using quantitative metabolomics to differentiate the diet-metabolites from those of the endogenous metabolites both qualitatively and quantitatively. Future work will improve confidence in our findings and provide additional biological context for quantified serum metabolites. In addition, it will be useful to determine whether serum metabolites correlate with tissue and lesion specific metabolites to better understand whether metabolic dysregulation is organ specific or systemic.

### Conclusions

The goal of this study was to investigate metabolic changes associated with development and progression of atherosclerosis using untargeted metabolomic analysis of NHP where genetics and environment can be controlled. We identified two heritable metabolites and found that abundance of some metabolites is sex-dependent. In addition, we identified 35 metabolites that correlate with total lesion burden and may serve as biomarkers for atherosclerotic lesion burden. This study provides a first insight into diet-induced serum metabolomic changes associated with CVDs. Future studies to validate these biomarkers should take into account sex-differences and influence of diet.

## Supporting information

S1 Fig Dual chemical derivatization (silylation) strategy to quantify metabolites using 2D GC-ToF-MS. A two-way Venn diagram displaying the unique and shared metabolites (180) between MTBSTFA (N-tert-butyldimethylsilyl-N-methyltrifluoroacetamide) and MSTFA (N-methyl-N-(trimethylsilyl)trifluoroacetamide with 1*%* trimethylchlorosilane), the two derivatization regents.
(TIF)

S2 Fig Network view of the quantified metabolites mapped onto KEGG pathways. KEGG-based metabolic pathways covered using the 2D GC-MS platform. The network view generated from MetaboAnalyst and modified using Cytoscape, displays the biological pathways covered using the metabolites quantified using 2D GC-ToF-MS used in this study. Nodes (yellow) are pathways and edges (lines) connect them for their relatedness.
(TIF)

S3 Fig Metabolite abundance differences in 32 baboon serum samples. Metabolite abundance differences (not significant, P, < 0.05) in the 32 baboon serum samples that were outliers, when comapred to itnernal standard (ribitol) used for the analytical runs. In the box-plots, the center lines show the medians; box limits indicate the 25^th^ and 75^th^ percentiles as determined; whiskers extend 1.5 times the interquartile range from the 25^th^ and 75^th^ percentiles, outliers are represented by dots; crosses represent sample means; data points are plotted as black dots. Widths of boxes are proportional to square roots of the number of observations.
(PDF)

S1 Table Clinical measures and fat score data for the 60 baboons at the start (0 yr) and end of 2 yrs HFHC time point. Columns represent: baboon number; sex, age at 2 yr experimental endpoint, triglyceride change (mg/dL), heart fat score, heart upper ridge fat score, heart coronary artery fat score, kidney fat score, change in body weight (kgs), change in glucose (mg/dL), LDL-c change (mg/dL), triglyceride at 2 yrs (mg/dL), body weight at 2 yrs (kgs), glucose at 2 yrs (mg/dL) and LDL-c at 2 yrs (mg/dL).
(XLSX)

S2 Table Lesion burden data for the 60 baboons at the end of 2 yrs HFHC diet challenge. (XLSX)

S3 Table Relative abundances of the quantified metabolites in the study across the two time-points (0 and 2 yrs) from 60 baboons as obtained using a 2D GC-ToF-MS platform using two derivatization strategies. (XLSX)

S4 Table Metabolites confidently identified and assigned as exposomal (environmental chemical, diet etc.) origin. Provided are 1^st^ and 2^nd^ dimension retention times (seconds), similarity matching scores against EI spectral libraries, signal/noise, unique (quantifier) mass, probability, retention time matrix.
(XLSX)

S5 Table List of metabolites showing differences based on diet and sex using a t-test. P-values reported are nominal (P, <0.05).
(XLSX)

S6 Table Heritability of metabolites showing N, h^2^, P-value associated with h^2^ and residual kurtosis for two diets (chow and HFHC). (XLSX)

S7 Table Pearson correlation matrices for male (upper) and females (bottom) baboons showing correlation between heritable metabolites, clinical measures and fat scores. (XLSX)

S8 Table Pearson correlation matrix for metabolites and lesion burden for the HFHC baboons. (XLSX)

S9 Table Metabolites displaying correlation with total lesion burden at the 2 yrs time point. Table is displaying R, slope, T-statistics, and P-values.
(XLSX)

## Abbreviations

2D GC-ToF-MS: two-dimensional gas chromatography time-of-flight mass-spectrometry
ANOVA: analysis of variance
BCAA: branched chain amino acid
CVD: cardiovascular disease
HCA: hierarchical clustering analysis
HDL-C: high density lipoprotein cholesterol
HFHC: high fat and high cholesterol
KEGG: Kyoto Encyclopedia of Genes and Genomes
LDL-C: low density lipoprotein cholesterol
m/z: mass-to-charge ratio
MeOX: methoxyamine hydrochloride
MSTFA: N-methyl-N-trimethylsilyl-trifluoroacetamide
MTBSTFA: N-(t-butyldimethylsilyl)-N-methyltrifluoroacetamide
NHP: non-human primate
PCA: principal component analysis
PLS-DA: partial least square-discriminant analysis
QC: quality control
