# Supplementary figures and images for: Analysis of serum changes in response to a high fat high cholesterol diet challenge reveals metabolic biomarkers of atherosclerosis
Source: PLoS One. 2019 Apr 5;14(4):e0214487. doi: 10.1371/journal.pone.0214487 (PMC6450610; doi:10.1371/journal.pone.0214487)

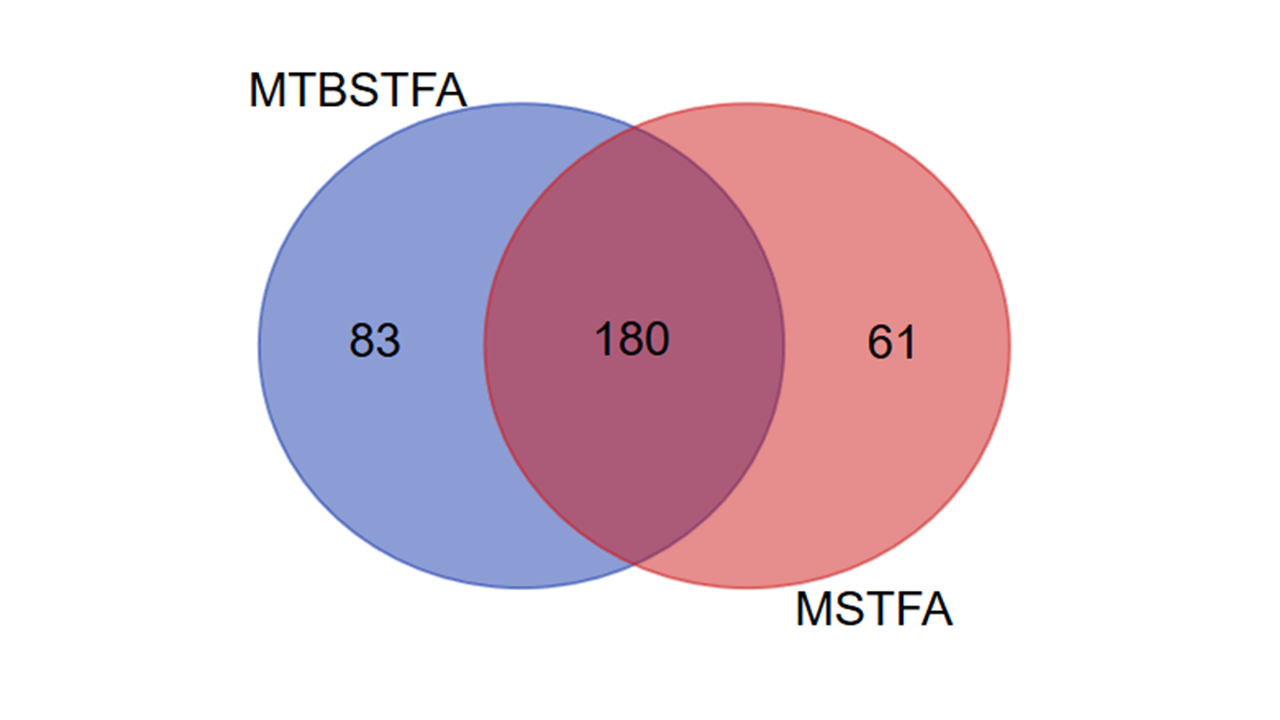

Supplement: S1 Fig — A two-way Venn diagram displaying the unique and shared metabolites (180) between MTBSTFA (N-tert-butyldimethylsilyl-N-methyltrifluoroacetamide) and MSTFA (N-methyl-N-(trimethylsilyl)trifluoroacetamide with 1% trimethylchlorosilane), the two derivatization regents. (TIF) [file pone.0214487.s001.tif]

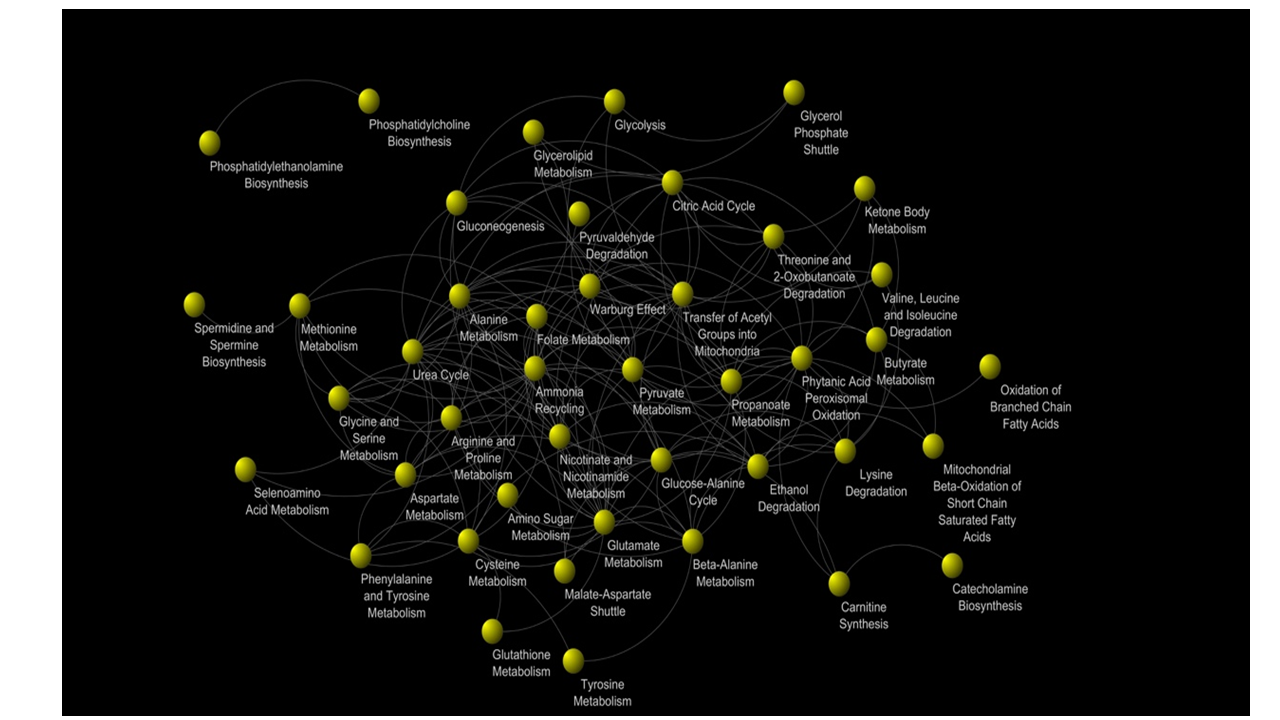

Supplement: S2 Fig — KEGG-based metabolic pathways covered using the 2D GC-MS platform. The network view generated from MetaboAnalyst and modified using Cytoscape, displays the biological pathways covered using the metabolites quantified using 2D GC-ToF-MS used in this study. Nodes (yellow) are pathways and edges (lines) connect them for their relatedness. (TIF) [file pone.0214487.s002.tif]
