# Supplementary material for: Analysis of serum changes in response to a high fat high cholesterol diet challenge reveals metabolic biomarkers of atherosclerosis
Source: PLoS One. 2019 Apr 5;14(4):e0214487. doi: 10.1371/journal.pone.0214487 (PMC6450610; doi:10.1371/journal.pone.0214487)
Supplement: S3 Fig — Metabolite abundance differences (not significant, P, < 0.05) in the 32 baboon serum samples that were outliers, when comapred to itnernal standard (ribitol) used for the analytical runs. In the box-plots, the center lines show the medians; box limits indicate the 25th and 75th percentiles as determined; whiskers extend 1.5 times the interquartile range from the 25th and 75th percentiles, outliers are represented by dots; crosses represent sample means; data points are plotted as black dots. Widths of boxes are proportional to square roots of the number of observations. (PDF) [file pone.0214487.s003.pdf]

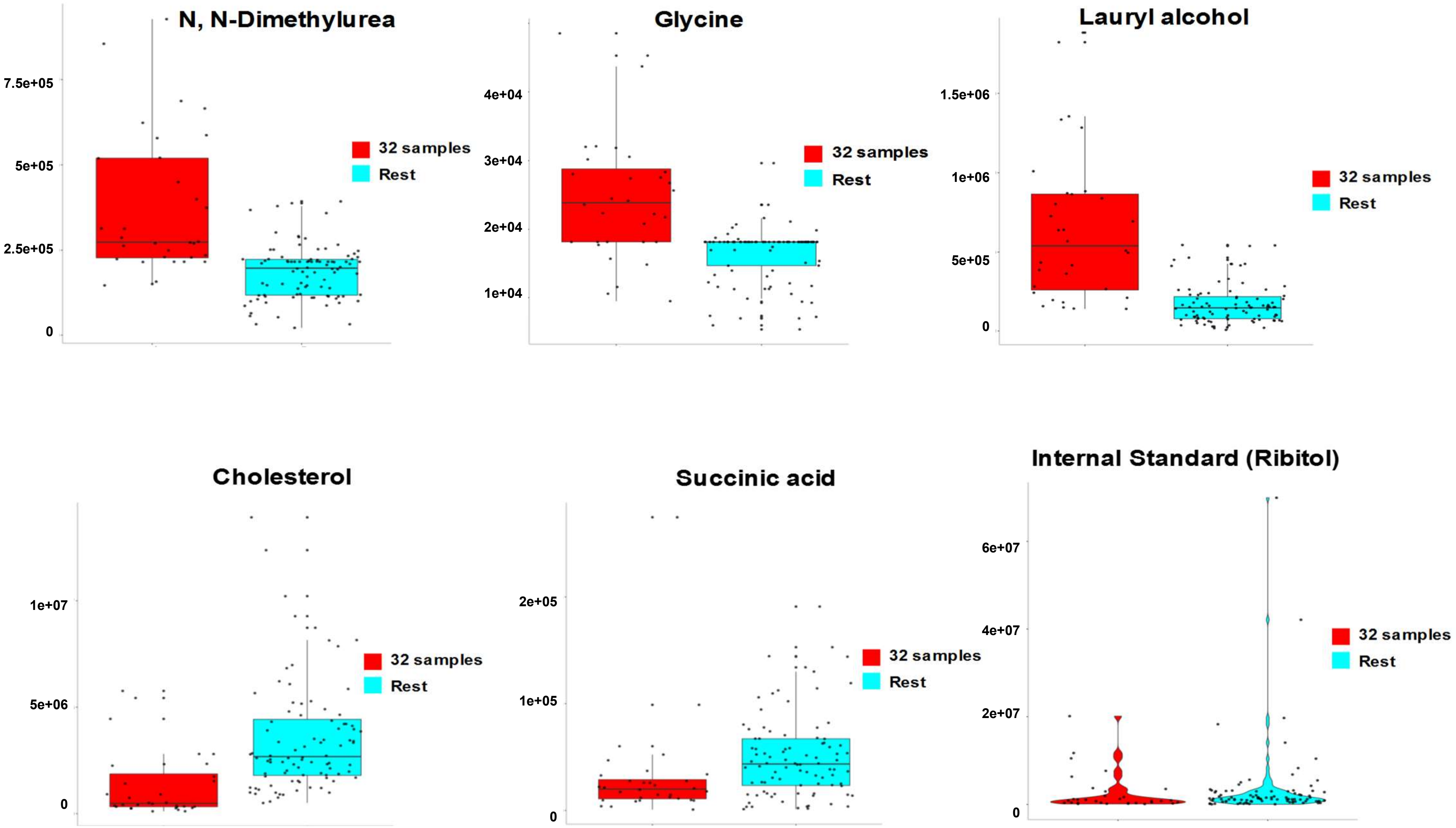

N, N-Dimethylurea
7.5e+05
5e+05
2.5e+05
0
32 samples
Rest
Glycine
4e+04
3e+04
2e+04
1e+04
Lauryl alcohol
1.5e+06
1e+06
5e+05
Cholesterol
1e+07
5e+06
Succinic acid
2e+05
1e+05
Internal Standard (Ribitol)
6e+07
4e+07
2e+07
